# A taxonomy of children and young people's social prescribing models: a multi-site implementation case study in England

**DOI:** 10.3389/fpubh.2026.1821348

**Published:** 2026-06-18

**Authors:** Vashti Berry, Siobhan Mitchell, Joe Coombes, Marcello Bertotti, Daniel Hayes, Lucy Cartwright, Kerryn Husk

**Affiliations:** 1 Public Health and Sport Sciences Department, University of Exeter, Exeter, United Kingdom; 2 Institute for Connected Communities, University of East London, London, United Kingdom; 3 Social Biobehavioural Research Group, University College London, London, United Kingdom; 4 Faculty of Health, University of Plymouth, Plymouth, United Kingdom

**Keywords:** children and young people, health systems, implementation, mental health, social prescribing

## Abstract

**Introduction:**

Social prescribing for children and young people (CYP) is expanding across England, yet there is limited understanding of how local services interpret and configure its core components—individual need, referrer, linking function and community activity—particularly for CYP presenting with mental health difficulties.

**Methods:**

Using a multi-site case study design, we examined five CYP-focused social prescribing pathways selected for their diversity of institutional anchors and implementation maturity. Data collection included pathway visualization with site leads and 34 semi-structured interviews with referrers, link workers, managers and practitioners. Thematic analysis was used to compare pathway structures and functions across cases to support development of an empirically grounded taxonomy.

**Results:**

Across sites, CYP accessed social prescribing through varied and uneven referral routes, with needs dominated by low-level mental health concerns amid gaps in statutory provision. Link worker roles differed substantially in embeddedness, remit and degree of mental health related support, often extending beyond traditional “linking” models. Pathways showed significant variation in institutional anchor points, eligibility criteria, linking functions and community asset connections. This variability enabled identification of key taxonomy dimensions, including institutional setting, referral pathway configuration, link worker function and community asset integration.

**Discussion:**

Findings show that CYP social prescribing is shaped by local context, unmet mental health need and flexible practitioner practice, resulting in diverse models rather than a standardized pathway. These differences have implications for commissioning and evaluation: rather than seeking uniformity, systems should support a ‘no wrong-door' approach and relational work, and evaluative approaches should prioritize implementation focused evidence over linear pre–post outcome measurement.

## 1 Introduction

**Figure 1:**
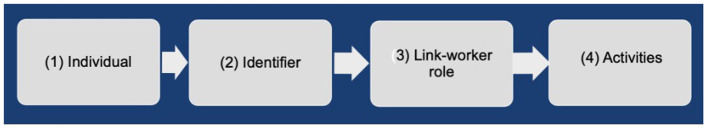
Core components of SP.

Definitions of social prescribing(SP) are various but are well summarized by Muhl et al. and describe the links from an ‘identifier' to community assets through an active link worker conversation for the purposes of health (1). These assets may include (but are not limited to) gardening programmes, physical activity sessions, cultural events or referrals for advice on debt. We have previously suggested a simplified ‘core components' model outlining the building blocks of social prescribing, being ‘individual need', ‘referrer', ‘linking function', and ‘community activities', as depicted in Figure 1 below (2).

Social prescribing is now embedded in core health policy in England (3) and has a role as a key component within the NHS personalized care agenda (4). Social prescribing is being rolled out across England, with over 3,500 link workers now employed through the Additional Roles Reimbursement Scheme (ARRS) in Primary Care. The NHS Long Term Workforce Plan commits to recruiting over 900,000 social prescribing link workers by 2036/37 (5). There is reach and scale, with new evidence showing 9.4 million consultations resulting in an SP code, and 5.5 million specifically resulting in an SP referral (6).

Social prescribing centers the social determinants of health, and recent epidemiological work has also shown that the model is associated with small improvements in patient experience and slightly better outcomes for population groups specifically targeted (7). There is evidence that social prescribing can improve physical and mental health and wellbeing, social contacts, and reduce service use and healthcare demand, though there are few studies with comparator, control or longitudinal designs and populations are often self-selected and not all studies report positive outcomes (7–12).

The UK Government-backed implementation of social prescribing was designed as a universal offer, with significant implications for rollout and scaling, but we know there are groups that access adult SP less often (8), that younger individuals are more likely to access SP through non-medical routes (13), and that existing inequalities may be perpetuated (14). Programmes proactively identify cohorts in need, including CYP, and there has been work around NHS England's CORE20 + 5 (15), but little dedicated research. In previous work, we have argued that there are reasons to believe that social prescribing for CYP holds promise as one way to deliver less stigmatizing community-based prevention for mental health. However, there are also reasons to think that behaviors, interactions with delivery organizations, and outcomes will all look different for this cohort (16).

The premise of this paper is that social prescribing-like pathways have the potential to offer CYP additional, preventative and alternative ways to manage symptoms associated with mental health difficulties. However, it is not currently clear how such pathways are implemented in practice, or how local delivery models interpret and configure the core components of social prescribing (the four building blocks outlined above). In this broad and loosely defined policy and practice context, the development of an implementation focused taxonomy of CYP-SP models is necessary to inform decision-making: enabling commissioning to better align with local system needs and partnerships and supporting the development of appropriate evaluation frameworks. A taxonomy that can be iteratively refined as evidence develops provides a foundation for aligning practice, commissioning and evaluation in CYP social prescribing.

## 2 Aims

Given the lack of coherent understanding about how social prescribing is operationalized for children and young people, our aim was to collect data that characterized and allowed us to describe these social prescribing (CYP-SP) pathways in practice. Specifically, we sought to develop an empirically grounded taxonomy of CYP-SP models by describing how the core components of social prescribing (Figure 1) are configured and implemented across sites in England. To achieve this, our CHOICES (*Children and young people's Options In the Community for Enhancing wellbeing through Social prescribing*) project examined how children and young people enter and move through different CYP-SP pathways, with a focus on those presenting with mental health difficulties.

Specifically, we set out to address two research questions:

- Research question 1 – Which individuals and services do CYP first present to when experiencing mental health difficulties?
- Research question 2 - How do young people with mental health difficulties move through CYP-SP pathways to access support?

## 3 Materials and methods

We adopted case study methodology to understand implementation of CYP-SP in five sites in England. Case study (17) is particularly suited to the investigation of complex service innovations, where interventions are shaped by organizational, professional and contextual factors and cannot be meaningfully separated from their setting. Each site constituted a bounded case of a locally configured CYP-SP pathway. By examining multiple cases selected for analytic diversity, we were able to compare how the core components of social prescribing were configured in different institutional and system contexts, supporting the development of an empirically grounded taxonomy of CYP-SP models.

### 3.1 Sampling / setting

Social prescribing is offered through virtually every Primary Care Network (PCN) in England, however as we note above this is an all-age offer and CYP are not specifically targeted. Dedicated CYP-orientated services are less numerous and have been set up in response to local needs and around existing funding structures that prioritize adult patients. No national registry of CYP-SP services exists and, while policy-driven, CYP-SP services are locally emergent and reflect different arrangements and maturity of partnership which makes systematic sampling impossible. As such, we adopted a theoretical and pragmatic case selection approach to maximize variation in services' implementation form. We made use of existing networks via sector partners (e.g., SPYN, NASP, StreetGames, SW SP Network) and hosted messages on the FutureNHS Social Prescribing Platform https://future.nhs.uk/ and other social media. These contacts enabled us to approach and recruit a selection of sites (*n* = 5) that were at different stages in their set-up/establishment (defined qualitatively), in different parts of the country, and interested to take part in the research.

Within each site, we undertook purposive participant sampling to capture perspectives from across the CYP-SP care pathway. Site leads were asked to identify and suggest key individuals; this typically included referrers, link workers, CYP practitioners and SP managers with responsibility for service oversight and commissioning relationships. We interviewed all consenting individuals who were involved in the immediate delivery and management of the pathway and a purposive subsample of referrers to reflect different referral routes and organizations. Sampling adequacy was determined through consideration of information power (rather than a numerical target) with the final sample judged to provide sufficient depth and breadth to support robust qualitative analysis (18). This approach enabled us to capture both operational and strategic perspectives on pathway functioning, referral practices and service integration.

### 3.2 Data collection and analysis

At each of these five sites, we conducted two waves of data collection during this phase of the project:

#### 3.2.1 Pathway visualization / mapping (N = 5)

At the outset of the project, two researchers conducted in-person site visits with each site lead and nominated staff or representatives (e.g., link worker/s, operating officer, lead nurse) who were highlighted as knowledgeable about the pathway, assessments and community asset partnerships. Using a semi-structured conversation topic guide designed to cover SP components (Supplementary material A), we worked with them to map the site's pathway against those known (or core) SP framework (Figure 1). Conversations were not video/audio-recorded but researchers took extensive field notes and from these derived a narrative description of the service and, in turn, pathway diagrams or maps (Supplementary material B). These were graphical representations (boxes detailing the key components of the service and arrows indicating links) of an individuals' journey through SP. These diagrams were shared with individual sites for further comment and refinement.

#### 3.2.2 Referrer and practitioner interviews (N = 34)

Each site was asked to suggest key individuals within the pathway, including referrers, link workers, CYP practitioners and SP managers. In the second wave, we invited these individuals to take part in a semi-structured interview to explore how children and young people progress through the care pathway and access social prescribing support for mental health concerns. Table 1 presents a breakdown of participants by site. Interviews were conducted virtually via Teams (topic guide in Supplementary material A; the topic guide was in part informed by pathway diagrams/maps from wave 1). Interviews lasted between 11 and 63 min (mean 36.5 min).

**Table 1:** Interviewee number and group by site.

Site	Number interviewed		
	Referrers	Link workers	Managerial
Site 1	4	1	3
Site 2	1	8	1
Site 3	2	1	2
Site 4	3	1	2
Site 5	2	1	2

Data from the interviews were analyzed thematically to examine how children and young people (CYP) first present with mental health difficulties and how they move through social prescribing pathways. Interviews were transcribed and analyzed inductively at the semantic level by three researchers. with coding supported by NVivo 14 (QSR International, Brisbane). Following the established stages of thematic analysis (19), the research team first familiarized themselves with the transcripts through repeated reading and note-taking. Initial codes were generated inductively and systematically across the entire dataset, with no pre-existing coding framework applied. Codes captured features of the data relevant to CYP mental health presentation and social prescribing pathways and remained close to participants' language and meaning. Coding was iterative with earlier transcripts revisited as new insights emerged. Codes were compared within and across transcripts to identify patterns of shared meaning, which informed the development of preliminary themes. These themes were reviewed in relation to the coded extracts and the full dataset to ensure internal coherence and clear distinction between themes and were refined through discussion and consensus within the research team. This process also involved considering relationships between themes, which informed the development of the final thematic structure and taxonomy dimensions presented in the findings. The taxonomy itself consists of domains that are second order constructs generated by the author team, using the first stage qualitative analysis, informed by the pathway diagrams and core SP components, and refined through discussion. Several strategies were used to enhance the rigor, consistency, and credibility of the qualitative analysis. Partial double coding (20%−30% of transcripts) was undertaken to support shared understanding of code meanings rather than to quantify agreement. Differences in coding were discussed within the research team, leading to refinement of code definitions, clarification of analytic boundaries, and more explicit articulation of underlying interpretations. Regular team meetings provided opportunities to review developing themes, question assumptions, and ensure that analytic claims were grounded in the data across participant groups.

Findings were further strengthened through triangulation, which involved the synthesis of data from multiple study components, including pathway mapping activities and interviews with referrers and practitioners. Triangulation was undertaken at the interpretation stage, following separate analysis of each data source, to build a more comprehensive understanding of how children and young people move through social prescribing pathways. This approach followed the triangulation protocol outlined by Farmer et al., whereby codes and emerging findings from each component were systematically reviewed alongside one another to identify areas of convergence, complementarity, and divergence (20). Through this process, themes derived from pathway mapping and interview data were compared, integrated, and refined to develop higher order themes that cut across data sources. This interpretive synthesis supported a more robust and nuanced account of shared mechanisms, experiences, and system level processes, while also highlighting how perspectives differed between referrers and practitioners. The triangulation process enhances the credibility of the findings by demonstrating how interpretations were informed by multiple forms of evidence rather than a single data source. Anonymity was maintained through grouping interviewees by professional role where possible and careful selection of illustrative quotations.

The development of the taxonomy was undertaken across three stages:

1. Within-case pathway specification: For each site, pathway visualization (see Supplementary material B) and interview data were synthesized to produce a detailed description of how young people entered, moved through and exited the CYP-SP pathway. These descriptions focused on institutional location, referral routes, link worker roles and community connections.
2. Cross-case comparison: Using a constant comparative approach, we systematically compared pathway structures and practices across sites to identify recurring patterns and points of divergence in how the core components were implemented. Differences were examined higher order dimensions identified that captured key sources of variation. These dimensions formed the basis of the taxonomy.
3. Refinement and validation: The emerging taxonomy was refined through repeat engagement with the data, including checking the fit of dimensions against individual cases and reviewing interpretations with the wider research team. Pathway diagrams and interview findings were used to test whether the taxonomy adequately captured both shared features and meaningful differences between models.

#### 3.2.3 Researcher reflexivity

The research team comprises university-based researchers situated within medical schools with backgrounds in child mental health research who were not working as practitioners, nor employed within social prescribing services or specialist mental health services during the study. As non-practitioner researchers, the team brought an analytical perspective focused on understanding pathways, access, and system level processes, while maintaining distance from day-to-day service delivery; at the same time, their positioning within university medical schools is likely to have shaped how evidence was identified, interpreted, and reported.

Alongside this academic positioning, team members also bring professional—and potentially personal—familiarity with mental health systems and care pathways, which may have shaped assumptions about how children and families navigate support. To address this, reflexivity was embedded throughout the analytic process and supported through collaborative and iterative practices, including team-based coding, double coding of a subset of transcripts, and regular analytic discussions. These discussions fostered critical examination of interpretations, consideration of alternative readings of the data, and explicit grounding of themes in participants' accounts, thereby supporting transparent and credible interpretation of findings. In recognition of our own positionality, we also sought to broaden perspectives through engagement with a range of stakeholders.

### 3.3 PPI and stakeholder engagement

Two groups were convened specifically for this project: a CHOICES Youth Advisory Group (YAG) and a Policy and Practice Board. The YAG was convened by StreetGames and made up of eight CYP, aged between 11 and 21 years, who had experience of mental health difficulties and/or being involved with social prescribing. The group met four times during the project, virtually and in-person, and the young people were involved in all aspects of the research, including designing and supporting delivery of community events in sites to gather input from CYP directly described elsewhere, Coombes et al., (in preparation), informing interview topic guides and supporting interpretation of the findings. In addition to gaining experience in research processes, StreetGames also ensured the YAG provided opportunities for members to gain wider experience specific to their personal goals (e.g., access to media software for art design, which was then used to design the community event poster invitations; attendance at a youth conference).

Our Policy and Practice Board comprised seven individuals with extensive experience in the field of CYP social prescribing policy and practice. This Board met twice a year during the project, and aimed to provide insight, feedback and support throughout the study, assist the research team to align project design with national and/or shared regional agendas, be a conduit for ensuring that the project and its findings were promoted in appropriate places, and provide advice on engagement with stakeholders and policymakers.

### 3.4 Ethics and informed consent

Ethical approval for the research project was granted from HRA (22/PR/0821), and the research was conducted in accordance with the Declaration of Helsinki. Initial meetings were undertaken with site leads by video-conference to explain the research commitment and obtain initial verbal consent to participate in the research. Site set-up visits were then conducted with each site in-person, where the study protocol and safeguarding procedures were discussed and written consent was taken. Individual consent was taken for each interview with practitioners, managers and referrers, including obtaining agreement to audio-record the interview for data collection purposes.

## 4 Results

### 4.1 Site pathway visualizations

Below, we describe some of the main or interesting features of the site pathways, however full pathway diagrams (based on the core components of SP) can be found in Supplementary material B. Site leads reported that the process of working through the pathway process with sites was useful from their delivery perspective, in addition to providing implementation data for the research. No site had previously conceptualized their programme in this way and finding common threads with other areas was considered beneficial, as well as enabling clearer communication of their offer to stakeholders.

Site 1 was more ‘policy traditional' in that it was based solely out of primary care, through GP referrals to link workers out of one primary care network with two linked youth organizations. Referral reasons were mostly orientated around mild to moderate mental health symptoms, such as anxiety, low mood, self-harm or anger. Ages ranged between 11 and 18. Of note, adult and CYP referrals to link workers were pooled and then triaged by a dedicated link worker team to separate the CYP referrals. Link workers were primarily community-based and initial appointments were conducted by phone, with follow up in person. A range of activities were linked to; limited by the rural location of the programme, not all CYP were linked to a community asset. The mean number of link worker interactions was 5.5 sessions.

Site 2 was a more devolved model with multiple entry points: through primary care, through the voluntary organization itself and through a school, with link worker involvement subtly different in each pathway. Cohorts are reportedly similar through each entry point, focused on mental health, emotional support, sexual health and similar. Ages ranged from 13 to 26. The programme reports all routes being more focused on emotional support rather than specifically linking to community activities. The mean interaction length was 6 sessions.

Site 3: one of the primary features of this programme was the very large range of entry points to the service, including CAMHS, third sector, other mental health agencies, schools, parents, and youth justice services. Ages ranged from 11 to 18 but this was considered flexible to reflect the gaps in existing provision. Link workers in this service were based in community groups and worked flexibly and were also able to attend activities with the CYP for up to three sessions if that was considered appropriate. Not all referrals ended with onward linking to community groups, but link worker support was sufficient. Re-entry to the service after ‘completion' was supported, usually through provision of a phone number or other contact details directly to the service.

Site 4 was a Higher Education College-based site where the main referral route is through that college, therefore the age range is 16–18 primarily. There are direct links and collaboration with the existing pastoral care team at the college, and this pathway extends the remit of that team through the addition of a community connector/link worker. Re-referrals are accepted, and as with other services described there is no stipulated need for onward referral to community when interaction with the link worker is sufficient. No limits had been set on number of contact sessions.

Site 5 was located in secondary care. There was a single point of entry and referrals were made by specialist clinical teams to the link worker and then discussed in weekly meetings dedicated to integrating link workers and teams on specific cases. The link worker was community-based and meetings held in public venues but also held space and periodic events/meetings on wards where appropriate. Re-entry was more informal than other settings, with contact maintained between link worker and CYP. Average contact was 4–6 sessions. The age range was stated as 11–18.

### 4.2 Interview data

Having described the pathways structures, we then analyzed qualitative data to add richness and detail to those descriptions and to address the two research questions more directly. Below we present these data structured around these research questions, split further into themes and subthemes that were constructed in analysis.

#### 4.2.1 RQ1 - Which individuals and services do children and young people first present to when experiencing mental health difficulties?

CYP present mental ill-health symptoms to different professionals, in different ways, at differing point in health trajectories. The sites involved in our study provided us with a range of perspectives. The referrers we interviewed included GPs, pediatric clinicians and nurses, social workers, pastoral staff in colleges, and college course teachers. Alongside this we also have the perspectives of link workers and health and wellbeing practitioners who worked within these services delivering social prescribing. Across these perspectives and services there are differences in how CYP access SP services, who is considered eligible for SP services, and how practitioners use SP as a ‘tool' to support CYP.

##### 4.2.1.1 Theme 1 - referral pathways

###### 4.2.1.1.1 Varied access

Point of access and demographic characteristics differed between sites, with different individuals being offered as the first point of contact. Referral routes varied widely across sites with more routes in providing greater and potentially more equal opportunities to access social prescribing. For example, in one site, GPs were the only option for referral into social prescribing, whereas others offered multiple routes such as GP, school, social work, self-referral, parent or carer, or referral by another professional.

…*self-referral, or it's a referral they have done with their parent because they have either been told about [removed] or they found it online and had a look and thought it might be appropriate to them…Other professionals and other charities and that kind of thing, will refer in as well if they think somebody is appropriate*. [LW, Site 2]

…*we get a lot of referrals from external agencies who are working with young people, so like Career Connect. We get referral directly from Prince's Trust. We get internal referrals from people who are struggling on curriculum courses, their attendance has dropped and different things. And it's sort of looking at a more holistic approach of how we can support them*. [Referrer, Site 4]

In those sites with fewer referral routes, such as GP only, referrers reflected that it may have implications for the level of severity in need that they are seeing. For example, a young person's level of need may be more severe by the time they come to see a GP, compared to when they could be identified in a school setting.

*I think that the last place a young person goes if they're feeling… they're struggling might be their GP … I think school has a lot of need … we're in contact with the teachers, and we know that there are young people who struggle and just wouldn't come and see us … So there's a puzzle there around whether the referral pathway's right at the moment with it being through us only at the moment*. [GP Referrer, Site 1]

Practitioners also picked up on differences in referral needs coming through different referral routes. In Site 2, where they have both GP and school referral routes, practitioners reflected on differences between the referrals received through each.

…*because of my PCN, because it's… it's… it's quite isolated, the… the young people that I see in the GP also go to the schools that I work in and vice versa, and I would say that in terms of the difference with young people, something that I definitely notice is that young people with an ASD diagnosis often come through the GP, and I work with thirteen to twenty-five, so… the schools that I work at don't have a sixth form, the school finishes at sixteen. So, anyone over sixteen comes through the GP as well. And often they come with different difficulties. When you go past sixteen the difficulties just change, so that's quite key as well, that there's older young people coming through the GP*. [Wellbeing Practitioner, Site 2]

##### 4.2.1.2 Theme 2 - link worker role and function

###### 4.2.1.2.1 Unmet need for mental health issues

Across our sites, the biggest need described for young people referred to social prescribing was low-level mental health need (e.g., anxiety, low mood, self-esteem and confidence issues) and social isolation. Social prescribing was described by some as a helpful additional service in addressing these issues, and for others this was viewed as something which ‘fills a gap' and acts as an alternative to referral to statutory mental health services such as CAMHS. There was positive description by referrers centered around how social prescribing increases capacity: reducing burden on GPs, preventing the need for significant medical input, and potentially having a preventative domino-effect on other services in the long-term.

###### 4.2.1.2.2 Filling a gap in mental health provision

Various different ‘gaps' were described including the threshold gap of other services; the limited services available between statutory and community; gaps in stepping up or down from statutory services (i.e., waiting periods for services such as CAMHS and limited options for support post-CAMHS intervention); and a general increase in the level of need for services creating a shortfall in options for young people's support.

*I guess, to be honest, we refer to secondary care less and less because the waiting times are so long, that it's almost pointless. It's almost like well by the time they've been seen, they've graduated, or they've left, or they're better, or I've got the answer elsewhere*. [GP, Site 2]

Many referrals into social prescribing were described as being for CYP with mental health issues who were unable to access other mental health services. Referrers described barriers to CAMHS referral, such as an onerous process, long wait to be seen, high threshold (and consequent referral rejection), and limited service offer, with GPs reflecting that instead of offering a CAMHS referral, they would offer social prescribing instead to a young person seeking support for mental health. There is also limited availability and consequentially long waiting times for other specific services, including counseling and school wellbeing services.

*There is definitely a massive gap in the things that we can provide children and young people, particularly with sort of complex emotional needs, but not necessarily meeting criteria for children and adolescent mental health. There is really no in between in terms of services. Neither sort of low level psychological first aid or just befriending services, there's just not very much around. There's a lot that falls back on schools providing things, and that's very variable depending on schools, and for the children who can't attend schools for a variety of health reasons or are struggling, it then… they're sort of stuck*. [Referrer, Site 5]

One Link Worker questioned if social prescribing would be necessary at all if there was a more appropriate mental health service in the middle.

*Definitely, there is a gap in the market, I suppose, for an extra role within the PCN then I wonder would they still want the social prescribers if they had that because we would get a lot less referrals I think at that point*. [Link Worker, Site 2]

###### 4.2.1.2.3 Mental health need beyond Link Worker remit

After the lack of clarity practitioners and referrers describe in where SP fits in terms of prevention and treatment and therefore who gets referred to SP, many who are referred were described as beyond LW remit. Many practitioners described a disconnect between the level of need young people who are referred present with and the remit of SP provision. There was also emphasis on how young people's social prescribing deviates from a traditional/adult SP model, in that the primary need is often mental health oriented.

Some Link Workers described a discrepancy between the role that was advertised, based on the adult model, and how they operate in their role with young people. Link Workers described being placed in a difficult position where they are faced with young people needing mental health support that is beyond their remit or training (e.g., counseling) but also feeling responsible because young people have nowhere else to go. It was felt that mental health support in the form of one-to-one talking and things like working on coping skills was more important than activities for most young people referred in, or that mental health needs are addressed first, before considering activities*:*

“*There was two cases... cases they referred that they were high risk, so I just... we just had to close, because it just wasn't appropriate for me to work with them.”* [Link Worker, Site 4]

*A lot of them aren't in a place to engage with activities. You know, like, in the community. They need that relationship first and the confidence-building and the work around how they feel first to then be able to engage with the activities … So a lot of the work is emotional, wellbeing support and the aim is to get to a point where they can engage with activities*. [Link Worker, Site 2]

A disconnect between the remit of SP services and the needs of young people being referred impacted upon the nature of support being provided and for some link workers led to a lack of clarity in their role and remit. High levels of mental health need resulted in LWs providing greater emotional support and signposting and less linking to activities.

*I found the job incredibly stressful when I started, incredibly stressful. I started during lockdown… the needs of young people it… you know I didn't feel there was a clear boundary of what we're supposed to be doing and what we weren't supposed to be doing*. [Link Worker, Site 1]

Additionally, referrers and practitioners described how sometimes the high level of need could be problematic in terms of engagement.

*I don't know if that's partly that when you are depressed, your self-esteem is very low and your self-confidence and what you feel you are entitled to is very, you know, “Oh, no, I don't deserve that”. And also, just that there is still some stigma around mental health problems, and so people feel like “Oh, I should just be able to pull myself together” or, you know, “I shouldn't be feeling this way, this is just me being weak” or, you know, especially if they come from a very traditional background*. [GP Referrer, Site 2]

#### 4.2.2 RQ2 - How do young people with mental health difficulties travel through the care pathway to access support?

Young people's journeys in social prescribing varied across our sites but throughout there were consistent themes around building trusting relationships, providing consistency in support, and the importance of communication, support, and visibility. These elements were described as key to ensuring that that the service was flexible to the requirements of CYP, and the presentation of the offer was clear and consistent for referrers and young people across a service.

##### 4.2.2.1 Building trusting relationships with young people

Link Workers described the importance of building trusting relationships with young people from the first contact onwards, with careful consideration made to explain the offer that is being made, including a description of what social prescribing is and its limits. It was described as important that this trust is developed with the referrer as well as the link worker and that the link worker is embedded into that system of trusted people:

*So, I think for young people it's just such a clear way, and they really trust and know their team at the hospital... I think that builds that trust straight away, you know, when a young person might be telling you something, they go oh, this doctor and I'm like oh yeah, I know that doctor, they're kind of like oh okay good, that's reassuring because we want to make it as easy as possible for people to engage and really focus on their health and what they should… what they could be doing, you know*. [LW, Site 5]

The first contact is made predominantly by phone call and is described as being used to start building trust and giving choice to the young person. Link workers emphasized the importance of taking time to build rapport at the beginning of the social prescribing journey.

*It's… a lot of what we do is about relationships, and so it's about trying to build a relationship early with somebody that just builds trust that just is clear around what we… who we are and what we are, and what we… we do… what do we offer*. [Manager, Site 1]

…*the young people were comfortable around me. If we met in a Costa or the library, it didn't look like a clinician or didn't look like someone they were… you know, had to be there with, it was more like… it just looked like we were kind of hanging out or, you know, it didn't look as… as scary as I think it could have been*. [LW, Site 5]

The importance of choice, flexibility and CYP-led decision-making is described throughout practitioner and referrer responses. Practitioners described giving CYP choices throughout their social prescribing journey. For example, flexibility in the location and format of sessions (mostly face-to-face, online, phone), the focus of sessions (exploring goals and aims and often focusing on preparing the young person to engage in SP e.g., through emotional support), the duration of sessions with rough remit but remaining flexible.

*I guess what works really well is like my flexibility with them. They don't just have to be at the college for me to meet them or for me to work with them. We can do it over Teams, we can do it at McDonald‘s, we can do it at a cafe, we can do it wherever. So I guess... I guess that's... that works better because obviously during the summer, even though most of them are on holiday, there's a few who can actually attend*. ? [LW, Site 4]

A central part of building rapport and getting to know a young person was exploring barriers. LWs described a key part of their role and the social prescribing process as understanding and exploring these barriers and then finding ways to support the young person to address or overcome them:

“*And… and another young person, you know, it was travelling on the bus that was a big issue there, so you know, they'd travel on the bus together to the local library.”* [Manager, Site 5].

…*try and find out if there's any barriers, so if it's transport, if it's money, if it's people even. I'm working with a young person, and she was actually targeted by a gang of young people, so she was attacked by six other young people, so a big thing for her is area and what people are going to be at that group. So that's like a barrier for her. So it's just kind of, like, navigating that*. [LW, Site 3]

##### 4.2.2.2 Providing consistency in support

For link workers, providing consistency and stability for a young person was central to their role. Often this involved being flexible in terms of the frequency or duration of sessions and holding onto cases until they had other types of support or engagement in place.

*So, I will try and meet with a young person consistently weekly because I think if a young person's in a space where they want to improve their well-being then there needs to be a kind of consistent checking-in and kind of moving forward with whatever they decide. So, I would try and see someone weekly, and we would gradually work out what they wanted to do*. [LW, Site 1]

This approach was often underpinned by the consistency and permanency of the Link Worker in post. The long-term presence of someone who was both consistent and embedded within the service was described by both managers and referrers as central to providing a successful SP service.

*We've not been doing it as long as we'd wish to, and I think that is one of the, I suppose for me, the key risks. A lot of those, that mapping exercise and those relationships are built with the person, so when a person leaves, and we find that within the wider programme, when the person leaves, those relationships do go with them. So, it is really difficult, and a lot of PCNs will go, you know, oh well just employ another person, but it does take a long time to get them up to speed with what's available in the community*. [Manager, Site 4]

##### 4.2.2.3 Interaction of services and pathways

Interaction of services and pathways was highlighted as integral to success, with sites emphasizing the importance of professional relationships and communication, support and visibility within this. Regular communication and the importance of building good relationships between different staff groups was described positively across sites, in the form of internal team meetings, multidisciplinary team meetings, and useful communication and support between Link Workers, including sharing knowledge with adult social prescribing teams.

In one site, the CAMHS team were described as particularly supportive, with primary care mental health workers offering both routine support and being available to help with any additional queries on demand. This was valued by the Link Workers involved and seems to be a useful factor, given the issues mentioned above with regard to Link Workers often working outside the social prescribing remit for mental health. Safeguarding of young people was key across sites, with Link Workers being clearly familiar with internal and external safeguarding procedures.

*Then we have the primary mental health worker from CAMHS that comes in and works with us once a month and we sit down. That is a smaller group usually, and she just again does a check-in and then asks about any cases and gives you advice on what she thinks you should do*. [LW, Site 2]

Communication was reported as less successful regarding GP referrers, creating barriers to referral or demonstrated by inappropriate referrals. GPs described wanting more information about what Link Workers provide in order to make referral decisions, with Link Workers sharing this view by stating that GP knowledge and awareness of social prescribing is limited, resulting in referrals being made that are not suitable for social prescribing, or young people not knowing what they have been referred for. In one site, it was described that GPs don't always provide a full picture about a young person and that young people can be accepted into social prescribing when their issues are too complex.

Link Workers described situating themselves in, or regularly revisiting, GP Practices or schools to remind them to refer into social prescribing. In some sites, this was described as positive and a way to raise awareness, while in others, challenges were highlighted. For example, GP Practices lacked the space to host Link Workers and without Link Worker visibility, social prescribing is forgotten, and referral numbers decrease.

In addition to raising numbers of referrals, this kind of connected system and communication was described as enabling a greater understanding of the service and better referrals to be made.

*I think that's a big thing as well, because obviously if they see you walking around working with young people, that kind of thing, it's more in their brain to think, oh, I will refer into that as well, whereas if they don't really see you, they don't really think of it as an option*. [LW, Site 2]

*I think there was some difficulty, I think especially at the start, getting the information out there or getting the service out there for the clinicians was a challenge. I think some of the clinicians were very, very excited, very… like, really for it, but then it just takes a little bit of time for them to then start seeing young people that fit the criteria, and… but that… I think that was a challenge we… I overcame because I was just always present and I would just drop into clinics and I would shadow clinics as well*. [LW, Site 5]

In Site 5, the LW described being fully embedded within the wider team. While it was challenging because SP was very new to the secondary care space, this approach provided opportunities not just for raising clinician awareness and boosting referrals, as described above, but also for ongoing communication with the referrer, supporting a feedback loop within the service and helping to mitigate challenges such as CYP engagement.

*So, she'd [LW] just… every now and again, just pop into our office and just update us on how she's been getting on and all the exciting things that she was doing with the patients and… or even if she was… sometimes she was having trouble getting in contact with them after initial referral and she'd update us on that, and… and there was one patient in particular who we… we had to help kind of contact as well, because she was really having a bit of difficulty with them. So, yeah, regardless [LW] was…was just keeping us in the loop and letting us know what was going on*. [Referrer, Site 5]

### 4.3 The taxonomy

Our process of creating pathway diagrams and qualitative analyses showed that while all sites contained the four core components of social prescribing (individual, referrer, linking function and community activity), the ways these were assembled, prioritized and operationalized varied substantially. These differences were not random: they reflected differences in local institutional anchors, service histories, professional relationships and gaps in mental health provision. By comparing the five pathways (shown in Supplementary material B) and integrating interview data on referral practices, link worker roles and community connections, we identified four higher-order organizing domains that together define the system configuration and operational logic of CYP-SP models: Institutional anchor; referral pathway configuration; link worker function; and community asset integration

These dimensions are intended as analytic constructs rather than fixed categories. Individual services may exhibit hybrid characteristics or shift position over time; nonetheless, across the five sites, consistent patterns were evident along each dimension, enabling systematic comparison as displayed in Table 2.

**Table 2:** Taxonomy of CYP-SP applied across sites.

Domain	Site 1	Site 2	Site 3	Site 4	Site 5
Institutional anchor	Primary care network (GP-based model with link to youth organizations)	Mixed model: Primary care + VCSE organization + School	Multi-agency: CAMHS, schools, VCSE, youth justice, MH agencies	Higher education college (pastoral care partnership)	Secondary care (specialist clinical teams)
Referral pathways	Targeted/MH presentations. Single gatekeeper (GP). Primary care adjunct/step-up MH support.	Broad eligibility but MH/emotional support-themed needs. Hybrid early/alternative support.	Broad and flexible eligibility. Highly open entry; multi-agency. System bridging/alternative pathway.	Broad/age-based eligibility. Multiple points of entry but all single institution. Early intervention support.	Highly targeted (clinical population). Clinician referral. Step-down support/clinical adjunct.
Link Worker (LW) Function	Brokerage – linking to activity. Limited additional support. Clinically anchored via GP referrals; community-based LWs operating between primary care and local youth organizations.	LW interaction and support (relational). Less emphasis on structured community linking. VCSE-based with negotiated links to GP and school; greater autonomy from clinical teams.	Linking to activity with additional (accompanied) support. No requirement for onward referral. Community-embedded LWs; strong autonomy; porous boundaries with statutory services.	LW interaction and signposting within the institution. Informal support, no requirement for onward linking. Embedded within statutory education setting (FE college); aligned with pastoral care team	Focus on MH support (adjunct to clinical care). Brokerage to community activities where appropriate. Integrated within specialist clinical teams; referrals clinician-led; LWs bridge ward and community
Community assets integration	Linking/community navigation. Referral-led model with established but arms-length relationships with community. Limited accompaniment.	Linking with relational preparation. Strong informal VCSE networks; negotiated access into community groups. Limited accompaniment but local availability.	Supported/accompanied model. Strong, sustained partnerships with community organizations; reciprocal relationships. Geography/access barriers actively mitigated by LW involvement.	Embedded/partnership-based (education-centered). Activities campus-based delivery reduces transport/safety barriers but lighter links with external/community assets.	Signposting/linking within clinical pathway. Brokerage following secondary care referral; limited accompanied engagement. Community links present but secondary to clinical care.

#### 4.3.1 Institutional anchor

The institutional anchor refers to where the CYP-SP pathway is primarily located and governed. This shapes referral flows, professional cultures, safeguarding processes and the kinds of young people who enter the service. Across our sites, five main anchors were evident: primary care–based (e.g., PCN-led models embedded in GP practices); education-based (e.g., colleges or schools with embedded link workers); voluntary and community sector (VCSE)–led; secondary care or hospital-based; and umbrella partnership models spanning multiple sectors. These anchors mattered; they structure referral flow, thresholds and the population reached, and the functions performed in supporting mental health. Primary care–based models tended to see young people later in their help-seeking journey and with higher levels of unmet mental health need, whereas education-based and VCSE-anchored models were able to engage young people earlier and more informally. Secondary care anchored models, by contrast, worked with young people already known to specialist services for particular health conditions (e.g., diabetes, sickle cell disease), often focusing on stabilization and transition rather than early prevention.

#### 4.3.2 Referral pathways

The second dimension captures how young people enter CYP-SP and how open or restricted that access is. Sites with multiple access points were more likely to reach CYP who would not otherwise present to GPs or mental health services, while single-route models tended to concentrate demand among those with more advanced or complex difficulties. This dimension therefore shapes who gets social prescribing and at what stage in their mental health trajectory. We identified three key features:

- Eligibility – whether services were open to all CYP within an age range, or targeted to speci'fic cohorts (e.g., those with mental health needs or those known to specialist services/with conditions).
- Number/type of entry points – these ranged from single-gatekeeper models (e.g., GP-only) to open entry systems allowing referrals from schools, VCSE organizations, clinicians, parents and self-referrals.
- Position in the wider care system – whether CYP-SP functioned primarily as an early intervention service/step-up pathway, as an alternative to statutory mental health services, or a step-down from secondary care.

#### 4.3.3 Link worker function

Across sites, link workers did not perform a single, standardized role although they all included brokerage in some form. Link worker (LW) function describes the way in which LWs supported young people with their health and wellbeing. This included:

- Linking to activity - following the ‘standard' social prescribing model where the LWs primary function is to link the individual to an activity within their community.
- LW interaction and signposting instead of linking - this function followed more of a youth work approach where the function of the LW was more relationship based and the linking was more informal.
- Linking to activity plus additional support - this dual function of the LW role involved going beyond simply linking the individual to an activity to providing additional support required to successfully engage in that activity. For example, traveling to the activity with a young person, or attending the activity with a young person for as long as was necessary to support their engagement.
- Focus on MH support - where a young person was not ready to engage in an activity and/or the mental health need was such that it would not be appropriate without first providing MH support, the LW role could focus solely on the function of emotional or MH support.

These functions were also impacted by the nature and rigidity of the LW ‘embeddedness' in different systems linked to the site (see Supplementary material, Table 1). There were implications that stemmed from being part of a primary or secondary care team and based between the GP or clinical setting and the community, or the converse where LW were based as part of VCSE organizations with entry into clinical settings negotiated, and relatedly where LW were based in statutory settings such as colleges and therefore not co-located with either clinical or community activity organizations.

#### 4.3.4 Community assets integration

The final dimension describes the extent to which CYP-SP pathways are linked/integrated into local community assets and how that enables them to connect young people to activities and resources in the locality. We identified three broad patterns across the five sites:

- Tailored signposting or community navigation models, where link workers identify and match options suited to the CYP interests/goals and encourage young people to attend independently.
- Supported or accompanied models, where link workers have strong community relationships and attend initial sessions with young people or provide intensive preparation and follow-up.
- Embedded or partnership-based models, where activities and support are closely aligned with, or delivered within, the CYP-SP service itself. Link workers might be embedded within referrer teams.

Both place (rural vs. urban) and geography, comprising distance to activities, availability of transport and safety, were major determinants of how far young people could realistically access community assets, meaning that relational and practical support often mattered as much as the availability of activities. In that context, the partnerships and relationships between CYP-SP services and local groups/resources were highly influential.

## 5 Discussion

Children and young people often present mental health difficulties in ways that differ from adults. Depressive disorders, for example, may manifest as irritability or somatic complaints rather than low mood, and symptom profiles such as anxiety, behavioral dysregulation or attention difficulties shift across developmental stages. These differences have important implications for how support pathways are configured and accessed by children and their families. The findings presented in this paper provide the first detailed cross-site analysis of the implementation of CYP-focused social prescribing (CYP-SP) services across five sites in England, selected to reflect breadth of practice. By including CYP-SP programmes at different stages of development and systematically mapping and analyzing their pathways using interview, visual and service-level data, we offer a structured account of how CYP-SP is operationalized in practice. This moves our understanding beyond high-level policy descriptions to provide an empirically grounded understanding of how social prescribing models are configured for children and young people within local systems.

A central finding is the variability of pathways across the sites. However, this is not random variation - it reflects how CYP-SP is being used to solve different system problems in distinct local contexts, including prevention and early intervention, improving access, managing waiting lists, providing step-down support from specialist services, or containing escalation to crisis care. Implementation is context-based and often driven by local social determinants of health; variation, therefore, represents functional adaptation rather than inconsistency, with important implications for delivery and evidence generation. Firstly, for delivery, efforts to reduce variation may be misplaced. Instead, there may be greater value in facilitating and supporting diverse entry points and routes through services. Rather than enforcing pathway standardization, best practice guidance (21) suggests focusing on core principles—named system leadership, safeguarding escalation routes, youth voice, protected workforce capacity and sustainable VCSE partnerships—while allowing local configuration to reflect population need. Secondly, what counts as high quality evidence for CYP-SP should reflect practice. Standardized pre–post outcome designs focused on core metrics are unlikely to capture how CYP-SP functions across heterogeneous system positions. Detailed implementation studies that examine how pathways operate, for which cohorts, and in what contexts — as exemplified by Bertotti et al. (22) — are likely to generate more meaningful and transferable insights.

We also observed significant differences between the adult/all-age models of social prescribing and CYP-SP. While variability in pathways is one distinguishing feature, differences were also evident in the community assets mobilized. CYP-SP offers did not consistently align with the five ‘pillars' commonly cited in adult SP (23); instead, provision was more generic, relational or, in some cases, centered on supportive engagement with no formal onward linkage. At the same time, there were striking similarities with the adult SP literature, which have implications for evidence generation. Challenges in collecting, collating and reporting consistent data across diverse pathways are common in CYP-SP, as they are in adult services (24), and potentially amplified by ethical standards and the role that other actors (parents, schools, clinicians) play as gatekeepers. As in adult SP (25), eligibility criteria were often locally determined and inconsistently implemented, making it difficult to specify clearly who services were intended for and complicating evaluation or comparison across sites. Additionally, maintaining contact between individuals, referrers and link workers through onward referrals to community assets is often complex and difficult, as is the understanding and communication around liability/responsibility throughout engagement. Finally, the findings reported here around link workers often engaging with CYP before and during onwards referrals links to work in the adult SP space around ‘holding' (26) and use of ‘discretion' by these roles (27).

The data also suggest that, in practice, CYP-SP is not consistently configured as an early intervention or preventative offer. Although prevention of mental disorder is frequently cited in policy narratives, eligibility criteria for early presentation are unclear and services tended to reply on diagnostic categories or established mental health conditions, rather than symptom-led identification or earlier precursors of distress. This positioning has important implications for both delivery and evaluation. It shapes what is recognized and recorded as need, what constitutes (and is measured as) “success,” and where CYP-SP is located along a prevention–intervention–recovery continuum (28). Given the policy and practice links between social prescribing and public health, there is scope for a more coherent articulation of how preventative cohorts might be engaged as programmes mature. Developing clearer strategies for including pre-diagnostic or sub-threshold presentations — and specifying intended outcomes for these groups — would strengthen both the coherence of CYP-SP as a preventative model and the evaluability of its impact.

We were able to produce a taxonomy of CYP-SP models from our case study sites. It is not exhaustive, nor does it rank or standardize services. Rather, it describes how (CYP-SP) is configured in practice across different institutional and system contexts. The taxonomy therefore provides a structured way of describing types of CYP-SP models, which cluster into distinct institutional and functional types, rather than treating social prescribing as a single, transferable intervention. Together, the four dimensions of the taxonomy capture the diversity of CYP-SP in real-world services. Rather than defining a single “best” model, the taxonomy allows commissioners, practitioners and researchers to ask questions such as: “What kind of CYP-SP model is operating here?” or “Who is it most likely to reach?” What counts as success depends on which CYP-SP model is in operation and attempts to impose uniform outcome metrics or standardized pathway designs are unlikely to work in practice.

Together, these analyses make a case for moving away from treating CYP-SP as a single intervention toward being understood as a collection of locally configured service models responding to different gaps in mental health and wellbeing provision. The findings highlight for commissioners the importance of moving toward commissioning for function within local systems. Given that services are addressing different gaps—ranging from early intervention to step-down support—commissioning should prioritize flexibility, multiple access points and integration across sectors. Investment in relational capacity, including sustained link worker roles and VCSE partnerships, is critical to engagement, particularly given the complexity and mental health needs of referred CYP. Finally, commissioning and evaluation frameworks should align, with expectations focused on implementation, reach and system contribution rather than standardized outcomes alone, reflecting the diverse roles CYP-SP services play across pathways.

## 6 Conclusion

This study provides the first detailed cross-site examination of CYP-focused social prescribing across five diverse settings in England. It demonstrates substantial heterogeneity in referral routes, service configurations and forms of community engagement when compared with adult or all-age social prescribing models. Crucially, this variation reflects contextual adaptation to local system pressures and social determinants rather than divergence from a single ideal model. These findings have important implications. For delivery, CYP-SP should be supported as a flexible, context-responsive intervention, with commissioning approaches that enable diverse entry points and relational modes of support while maintaining fidelity to core principles. For evaluation, frameworks must move beyond linear pre–post outcome measurement to incorporate implementation analysis, system positioning and contribution to outcomes across different cohorts. As CYP-SP programmes mature and scale, clearer articulation of their preventative, therapeutic and system-bridging functions will be needed to ensure both accountability and sustainable development.

## Data Availability

The raw data supporting the conclusions of this article will be made available by the authors, without undue reservation.

## Glossary

CYP, Children and Young People (can refer to anyone aged 0-24 years though services enforce different age thresholds) SP, Social Prescribing; NHS, National Health Services (publicly funded healthcare system in the UK); CHOICES, The ‘CHildren and young people's Options In the Community for Enhancing wellbeing through Social prescribing' study: https://arc-swp.nihr.ac.uk/research/projects/cyp-choices/; YAG, Youth Advisory Group; LW, (Social Prescribing) Link Worker (connecting people to community-based support activities and services); CAMHS, Child and Adolescent Mental Health Service (publicly funded mental health services in the UK); PCN, Primary Care Network (Groups of GP practices working together with community mental health and social care providers to deliver integrated services to local populations); VCSE, Voluntary Community and Social Enterprise sector; FE College, Further Education Colleges provide vocational technical and academic courses for students aged 16+ after secondary school in the UK.
