# Supplementary material for: A taxonomy of children and young people's social prescribing models: a multi-site implementation case study in England
Source: Front Public Health. 2026 Jun 18;14:1821348. doi: 10.3389/fpubh.2026.1821348 (PMC13323003; doi:10.3389/fpubh.2026.1821348)
Supplement: Supplementary file 1 [file Table_1.docx]

**Supplementary Material A - Topic Guides**

**Topic guide: Pathway Mapping**

1. **Mapping of services/pathways**

As part of our work we would like to ‘map out’ your service and the pathways within it – this will help us to understand how CYP are referred into and move through SP services. This will involve helping us to understand who the key people are in your service and how CYP come into and move through your service.

This will involve a discussion with the research team (which we will do as part of this meeting) and we will pull together an outline/diagram of what we have understood about your service and send it back to you to ‘sense check’.

**Outline plan for mapping of services and pathways and discuss**

Can you talk us through how a young person…

1. Gets referred to your service (who/how are they referred?) and what for? (i.e. MH specific or more broad? Are there different ‘pathways’ within your service for YP referred for different reasons?)
2. What is the geographical boundary/catchment area for your service? Do you have a map?
3. Once they are referred to your service, what happens to that YP? Who do they speak to? What options are they provided with? What support do they receive?
4. If there is a link worker role, who fulfils this role?
5. What kind of contact does the YP have with the link worker? (i.e. amount of time/appointments)
6. What activities do they have access to and at what frequency and duration? Log/database of activities? How do LWs source ideas/activities? Do CYP always get linked to an activity/asset? Or are there times where this does not happen?
7. What happens to the YP after this?
8. Have we covered all the key people CYP have contact with on their ‘journey’ through your service? Any other key people we should know about?
9. Explore level of need of CYP

**Topic guide: Practitioners and referrers**

If YP not ready to engage or already involved in activities – LW provides emotional support and coping strategies

An interview for practitioners and referrers (GPs, link workers etc) to help us understand how referrals are made and who makes them.

Time allowed: 1 hour

**Aims of the study**

We are interested in how young people connect with activities for wellbeing in their community and what kind of support they have along the way.

**Exploration of pathway**

1. **Can you tell us a little bit about your role in SITE#**

Prompts:

How long have you been in the role? What ages do you work with? CYP only?

How do you engage with CYP?

1. **What does social prescribing mean to you in your area? How would you describe it?**

Prompts:

What support is available to CYP? How do they engage with it?

*For GPs:* What's your understanding of SP? What type of CYP is this referral route helpful for? How do you utilise this as a 'treatment'/referral option? What types of CYP would you refer?/What makes a 'suitable' SP referral?

1. **How does social prescribing fit into your role?**

Prompts:

How does your role involve SP, specifically with CYP? How much of your role involves SP?

To what extent do you feel SP is a useful referral route for the types of CYP patient you see? What are your other options (if any) for young people in terms of referrals?

**3b. Can you tell me a bit about your experiences of social prescribing, specifically with CYP?**

Prompts:

When do you refer a CYP to social prescribing? How do you make this decision?

What makes a suitable SP referral/what types of CYP do you refer?

*For LWs:* What sort of activities can you connect CYP to in your community?

Prompts:

What do community assets look like in your area/how do you find out about them? i.e. Do you work with a cultural broker/community builder or any other community members to understand more about activities in your area?

What level of need do you see? Are there particular people / services who you get more referrals from / work more closely with? And conversely any you don’t but feel you might be able to work more closely with? Is SP regularly used and in what way?

1. **What does the referral process look like for young people engaging with social prescribing?**

Prompts:

Who is the first point of contact? Who is responsible for referring them to SP? Who connects them with the activity?/Is there a linking function/role?

Who has contact with CYP during the process?/frequency/duration of contact

How long is the process from referral to starting the activity?

Do young people have a say in what activity to engage with/What options did they have?

How are decisions made about this? (shared?)

Are there situations where you don’t link the YP with an activity/service? Why would that be the case?

*For GPs:* What's the process? Who completes the referral? Do you know how to refer? Is it clear? What is your role in this pathway/referral process? What information do you provide on referral? (what informs LWs when they 'triage' referrals)

1. **How do you support young people through this process? (not relevant for GPs)**

Prompts:

What support and/or training is available?

How much awareness/knowledge do you have about SP in your area?

How well supported do you feel?

Has this changed during the pandemic?

Can you give us a specific example of a pathway for a CYP?

1. **Do you have any views on how the SP process / pathway in your area is working particularly well? And / or any thoughts about difficulties or opportunities for development?**

Prompts:

Strengths/opportunities for CYP SP in their area?

Number of referrals? Ease of referral process? Usefulness of SP as a referral option?
