# Supplementary material for: A taxonomy of children and young people's social prescribing models: a multi-site implementation case study in England
Source: Front Public Health. 2026 Jun 18;14:1821348. doi: 10.3389/fpubh.2026.1821348 (PMC13323003; doi:10.3389/fpubh.2026.1821348)
Supplement: Supplementary file 2 [file Table_2.docx]

**Supplementary material B – Pathway Diagrams**

GP surgery refer via adult SP organisations who then check age and then send YP details onto either SPACE or YoungDevon

LWs are ‘floating’ – sometimes in surgeries but spend majority of time in community

LWs tend to meet with CYP on a weekly basis for around six hour long session, but this can be flexible.

*If 18, GP discretion whether to refer to YP or adult services

LWs refer back to GP with signposting advice if outside age range

If YP not ready to engage e.g. high MH need – LW provides emotional support and coping strategies

**After SP finished**

- 1-2 additional sessions can be offered in very limited circumstances (i.e. re-referral)
- LWs may follow up with a text but this depends on the individual.
- There is an ‘open door’ for YP once involved in the service

If YP not ready to engage or already involved in activities – LW provides emotional support and coping strategies


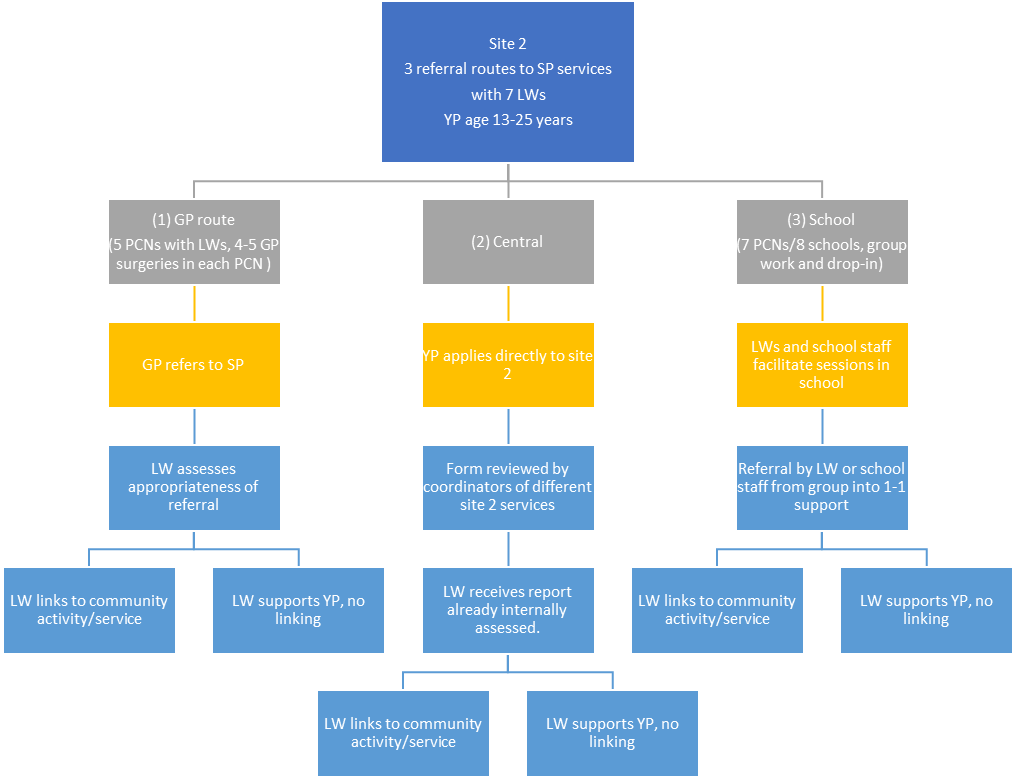


Main source of referrals

Two PCNs offer HWB practitioners

Recommenced their central LW post following funding from Council so they can continue to support young people from anywhere across [area], without the restriction of a PCN area.

Other points of contact:

Central pathway - coordinators may communicate with YP or invite them along to wellbeing Wednesday to meet with a health and wellbeing practitioner

Schools – depends on referral route – YP may have contact with pastoral lead before linking with LW.

GP route – may have contact with GP or care coordinator before linking with LW.

**After SP finished**

- Open access Wellbeing Café – delivered by Health & Wellbeing Team
- Can come back into SP (re-referral)

Contact with LW every 2 weeks. Up to 3 months of support ~ 6 sessions but not limited to this, can be flexible

Referrals received from the following:

- Social Work & Early Help Teams
- CAMHS
- NHS Social Prescribers
- Our own Link Workers
- Carers/Parents
- Schools
- Youth Services
- School nurse
- School Attendance officers (based in LA)
- Nurse Practitioners & GPs
- Project Choice
- Youth Justice Service
- Alliance

Hybrid governance model (YP SP steering group) means members from services/organisation know how to contact SM or LW to refer.

- LWs proactively embed themselves within local groups to gain insight and familiarity, which helps determine the groups’ suitability for CYP.
- The LWs will take young people along up to 3 times to a club/session and this could be longer if Link Workers are supporting them to get the bus. Young people often choose more than one activity so this would be the same support for access to other sessions.

LWs proactively embed themselves within local groups to gain insight and familiarity, which helps determine the groups’ suitability for CYP.

It is driven by young people but LWs try to see young people a minimum of one session per week. The Link Workers will take young people along up to 3 times to a club/session and this could be longer if Link Workers are supporting them to get the bus.  Young people often choose more than one activity so this would be the same support for access to other sessions.

LWs tend to meet with CYP on a weekly basis for around six hour long session, but this can be flexible.

**After SP finished**

- If a young person wants support after they have exited a referral does not need to be completed, there is no need to completed another referral.

*Mainly this college age range, although could be adults of any age over 18

Referral back to pastoral team if any safeguarding concerns

**After SP finished**

- Can be re-referred into the service

Physically sat together in the Learning Zone

LW meets with CYP roughly every 2 weeks for 4-6 sessions, but this is flexible.

**After SP finished**

- Follow-up after a month.
- No formal re-referral process but YP could have another session e.g. if they wanted to try something else
- Feedback requested from YP via email
