# Supplementary material for: A taxonomy of children and young people's social prescribing models: a multi-site implementation case study in England
Source: Front Public Health. 2026 Jun 18;14:1821348. doi: 10.3389/fpubh.2026.1821348 (PMC13323003; doi:10.3389/fpubh.2026.1821348)
Supplement: Supplementary file 3 [file Table_3.docx]

|  |  |  | **LW embeddedness** | | **LW function** | | | |
| --- | --- | --- | --- | --- | --- | --- | --- | --- |
|  |  | Part of p/c or s/c team  Floating (community based but sometimes based in GP surgeries/other clinical settings) | | Not part of p/c team not co-located  (community based e.g., embedded within local groups/centres/schools) | Linking to activity | LW interaction and signposting instead of linking* | Linking to activity plus additional support e.g., attending with YP | Focus on MH support where more appropriate |
| **Models of SP (pathway)** | Signposting only (more informal referral e.g., school) |  | | Site 4 | Site 4 | Site 4 |  |  |
|  | Link worker model only (GP /clinical > LW) – single referral route | Site 1, Site 5 | |  | Site 1, Site 5 | Site 1, Site 5 |  | Site 1 |
|  | Flexible, Holistic (multiple supported routes and deep engagement from the LW) |  | | Site 2, Site 3 | Site 2, Site 3 | Site 3 | Site 3 | Site 2 |

Supplementary Table 1. Link worker embeddedness and function across sites

*Note: p/c refers to primary care. s/c refers to secondary care.*

**More similar to Youth Work role*
